# Connexins and Integrins in Exosomes

**DOI:** 10.3390/cancers11010106

**Published:** 2019-01-17

**Authors:** Motomu Shimaoka, Eiji Kawamoto, Arong Gaowa, Takayuki Okamoto, Eun Jeong Park

**Affiliations:** 1 Department of Molecular Pathobiology and Cell Adhesion Biology, Mie University Graduate School of Medicine 2-174 Edobashi, Tsu-city, Mie 514-8507, Japan; a_2.uk@mac.com (E.K.); arong-g@doc.medic.mie-u.ac.jp (A.G.); epark@doc.medic.mie-u.ac.jp (E.J.P.); 2 Department of Disaster and Emergency Medicine, Mie University Graduate School of Medicine 2-174 Edobashi, Tsu-city, Mie 514-8507, Japan; 3 Department of Pharmacology, Faculty of Medicine, Shimane University, 89-1 Enya-cho, Izumo-city, Shimane 693-8501, Japan; okamoto@med.shimane-u.ac.jp

**Keywords:** connexin, pannexin, integrin, exosomes, crosstalk, cancer, inflammation

## Abstract

Connexins and integrins, the two structurally and functionally distinct families of transmembrane proteins, have been shown to be inter-connected by various modes of cross-talk in cells, such as direct physical coupling via lateral contact, indirect physical coupling via actin and actin-binding proteins, and functional coupling via signaling cascades. This connexin-integrin cross-talk exemplifies a biologically important collaboration between channels and adhesion receptors in cells. Exosomes are biological lipid-bilayer nanoparticles secreted from virtually all cells via endosomal pathways into the extracellular space, thereby mediating intercellular communications across a broad range of health and diseases, including cancer progression and metastasis, infection and inflammation, and metabolic deregulation. Connexins and integrins are embedded in the exosomal membranes and have emerged as critical regulators of intercellular communication. This concise review article will explain and discuss recent progress in better understanding the roles of connexins, integrins, and their cross-talk in cells and exosomes.

## 1. Introduction

Connexins (Cxs) [1,2,3] and integrins [4,5,6] are two structurally and functionally distinct families of membrane proteins that have been shown to play apparently different biological roles in intercellular communications, as detailed in the sections that follow. Briefly, Cxs primarily function as an integral component of channels at the gap junctions, where they mediate inter-cellular exchanges of small molecules [1,2,3], whereas integrins primarily function as cell-adhesion molecules that mediate cell-to-cell and/or cell-to-extracellular matrix adhesive interactions [4,5,6]. Several important modes of crosstalk between integrins and Cxs have been identified [7,8,9], underscoring the important collaborations between these two distinct membrane proteins in regulating cellular functions in both health and diseases, including cancers. Recently, investigations of Cxs and integrins have expanded to exosomes, the lipid bi-layered nanoparticles secreted from cells that are now recognized as a new biological means for intercellular communications under normal physiological and many patho-physiological conditions, such as cancers [10]. Exosomes display many membrane proteins—including Cxs [11] and integrins [12]—that originate from source cells. However, the functions and biological implications of exosomal Cxs and integrins remain little understood. Here, we aim to review recent progress in understanding how Cxs and integrins function in exosomes, and will then theorize how they might crosstalk in exosomes.

### 1.1. Connexins

Cxs are the family of channel-building proteins that span the membrane four times and that consist of two extracellular loops and one cytoplasmic loop, thereby comprising both the amino and carboxyl termini in the cytoplasm [1,2]. In humans and mice, more than 20 Cx proteins have been identified [2]. Cxs are assembled with hexamers, allowing the formation of Cx-containing gap junction channels and hemichannels. In this way, they are involved in the transmembrane transport of ions and small molecules between the cytoplasm of two attaching cells in the case of the Cx-containing gap junction channels, and between the cytoplasm and the extracellular space in the case of hemichannels [2,3]. Cxs also carry out biological functions independent of the Cx-containing gap junction channels and hemichannels [13]. For example, the ectopic expression of the connexin 43 (Cx43) carboxy-terminal cytoplasmic tail has been shown to be sufficient to mediate, through the Cx interactome, some of the Cx-mediated cellular functions, such as the regulation of cell growth [13,14]. The ability of Cx-containing channels to dynamically open and close is regulated by several distinct mechanisms, including protein phosphorylation, divalent cations, membrane potentials, and redox molecules [15]. In addition, several cytoplasmic proteins (e.g., Ezrin, α-tubulin, zona occludens-1 (ZO-1)) directly interact with Cxs, thereby regulating the activities of both channel and non-channel functions [1,13].

The diverse and complex biological functions of Cxs in cancers have been demonstrated [3,13]. Cxs exhibit either anti-tumorigenic or pro-tumorigenic activities, depending on the Cx subtypes, cancer origins, and cancer progression stages [3,13]. The anti-tumorigenic activities of Cxs are explained at least partly through the suppression of the cell cycle, whereas the pro-tumorigenic activities are explained through the promotion of cell migration [3,13]. The crosstalk with integrins and exosomal Cxs would provide a clue to better-explain the diverse activities of Cx in cancers.

### 1.2. Integrins

Integrins comprise the family of cell adhesion molecules that mediate cell-to-cell and cell-to-extracellular matrix interactions across a wide range of physiological and pathophysiological settings, such as cell adhesion and migration [4], cell differentiation and proliferation [16], organ development and tissue regeneration [17], mechano-transduction [18], inflammation [19], and cancer metastasis [20]. Integrins are heterodimeric membrane proteins consisting of α and β subunits [4,5] and comprise an integral component of integrin-adhesion complexes at focal adhesions, in which they play a key role in mediating bi-directional transmembrane signals [18,21]. Both integrin subunits are type I transmembrane proteins that span the membrane, with the extracellular amino terminus and the cytoplasmic carboxyl terminus. The amino termini of the α and β subunits are non-covalently associated with each other, thereby forming αβ integrin heterodimers [4,5]. In humans and mice, 18 integrin α subunits and eight integrin β subunits have been identified, thus comprising at least 24 different integrin heterodimers [4,5]. The most prominent feature of integrins is their ability to transmit bi-directional transmembrane signals via global conformational changes [5,18]. The extracellular domains of integrins undergo global conformational changes that lead to a rapid increase in ligand-binding affinity [5]. This occurs in response to intracellular signals elicited by the activation of other receptors, such as chemokine receptors and growth factor receptors [4,5,6,22]. Conversely, ligand binding to the extracellular domains of integrins enforces the stabilization of high-affinity conformations that sustain signaling to the cytoplasm, thereby modifying cellular functions including proliferation, metabolism, and migration [4,5,6,22]. Integrin-mediated modifications of cellular functions play an important role in many pathologies, such as in the chemo-resistance of cancer cells [23].

### 1.3. Exosomes

Exosomes are nano-sized biological particles secreted from virtually all cells, and constitute a subset of extracellular vesicles. As exosomes carry various bioactive molecules, such as enzymes, cytokines, eicosanoids, and small RNAs, they play critical roles in intercellular communications, both in healthy individuals and in those afflicted with diseases [10]. Among the various types of extracellular vesicles, exosomes are unique in that they are formed and secreted by the cellular endosomal pathway [24]. Of note, parts of internalized integrins [25] and Cxs [26] are directed to early endosomes in the endosomal pathway. They are subsequently sorted into different membrane trafficking routes to include the following: recycling back to the plasma membrane; forming late endosomes followed by degradation in lysosomes; and forming late endosomes followed by integration into exosomes in the multi-vesicular body. The latter are eventually fused to the plasma membrane, resulting in the release of exosomes into the extracellular space.

## 2. Integrin-Connexin Cross-Talk in Cells

The Cx-mediated cell-to-cell interaction that occurs via gap junction-mediated intercellular communications (GJIC) is known to be affected by integrin-mediated cell-to-extracellular matrix interactions [27], which serves as a good example of Cx-integrin crosstalk. To date, several studies have shown multiple cases of Cx-integrin crosstalk in various contexts. The mechanistic basis underlying the crosstalk between the two membrane proteins has been classified into at least three distinct modes, as shown in the following sections.

### 2.1. Direct Physical Coupling Via Lateral Contact

**Figure 1:**
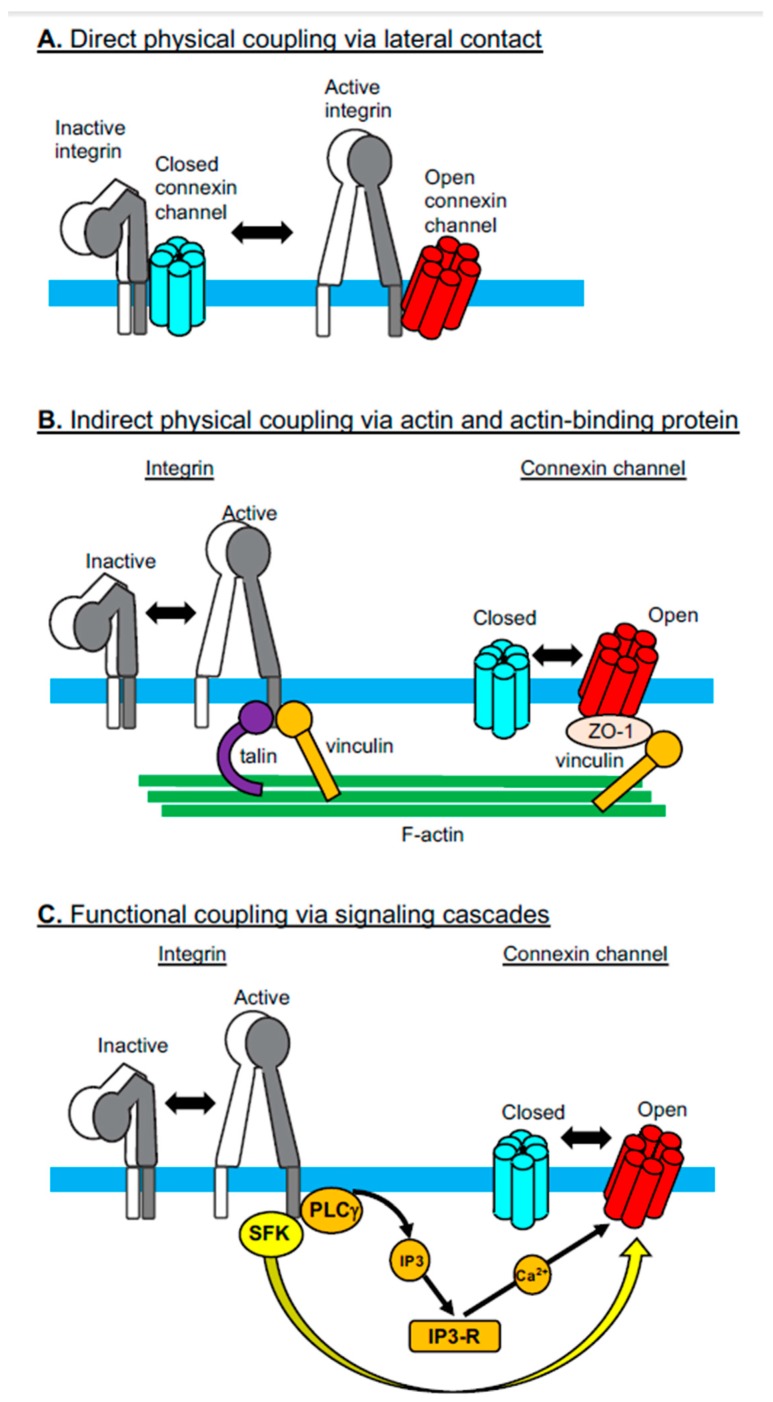
Different modes of cross-talk between integrins and Cxs. Three different mechanisms have been reported that explain how the activation-dependent conformational changes observed in integrins regulate the opening/closing of Cx channels: (**A**) direct physical coupling via lateral contact, (**B**) indirect physical coupling via actin and actin-binding proteins, and (**C**) functional coupling via signaling cascades. SFK: Src-family kinases; PLCγ: Phospholipase Cγ; IP3: Inositol-1,4,5-triphosphate.

The activities of integrins are regulated not only by their interactions with cytoplasmic adaptor proteins, such as talin and kindlin [28], but also by their lateral associations with other membrane proteins, such as members of the tetraspan superfamily of membrane proteins (e.g., CD9, CD151, Tspan12) [29]. Cx43 has been added as a new member of the membrane proteins that directly and laterally associate with integrins [7]. Jiang and colleagues have shown in osteocytes that the Cx43 hemichannels directly interact with integrin α5β1 [7] (Figure 1A). Notably, the presence of shear stress elicits intracellular PI3K signaling, thereby leading to integrin activation. Activated integrin α5β1 undergoes conformational changes, which enhances its physical interactions with Cx43. Enhanced physical interaction between the integrin and the Cx induces the opening of the hemichannels. A sequel study by the same group has shown that shear stress-induced AKT activation phosphorylates both Cx43 and integrin α5β1, thereby further facilitating not only the physical interaction between Cx43 and the integrin, but also the opening of the Cx43 hemichannels [30]. This physical coupling of integrins and Cxs suggests an underlying structural mechanism by which shear stress induces the transmembrane communication of small molecules, including anabolic factors [7]. Physical association with integrin α5β1 could stabilize the Cx protein, which may explain why transgenic mice expressing mutant-truncated integrin α5 lacking the specific residues to interact with Cx43 exhibit a reduced expression of this Cx [31]. However, a contrasting result was reported in cardiomyocytes that were differentiated in vitro from β1 integrin-deficient ES cells; in this setting, Cx43 expression was enhanced in the absence of β1 integrin [32]. It remains to be seen whether these contradictory results stem from different integrin manipulations (i.e., partial deletion vs. complete deletion), although Wnt signaling, activated secondarily to the β1 integrin deficiency, might be involved in the upregulation of Cx43 expression [32].

### 2.2. Indirect Physical Coupling Via Actin and Actin-Binding Proteins

In addition to the direct lateral interaction of Cxs with integrins, these two families of membrane molecules are inter-connected via the network of the actin cytoskeleton and actin-binding proteins (Figure 1B). Integrins are connected to actin via several different actin-binding proteins (e.g., talin and vinculin), which directly bind to cytoplasmic integrin domains [33]. By contrast, Cxs are connected to actin via the ZO-1-vinculin complex, in which ZO-1 and vinculin bind to Cx and actin, respectively. In this way, vinculin is involved in both integrin-actin and Cx-actin interactions, thereby playing a pivotal role in the cytoskeletal linkage that inter-connects Cxs and integrins [8]. This suggests a model in which gap junction Cxs at the intercalated discs are mechanically linked to integrins at the focal adhesions via the actin cytoskeleton and actin-biding proteins, such as vinculin, ZO-1, and talin [8]. Cx and integrin protein expression has been shown to be reduced during the conditional deletion of vinculin in cardiomyocytes [8]. In addition, pharmacological inhibition of a microtubule cytoskeleton that supports an indirect linkage between Cxs and integrins was shown to alter the distribution and expression of Cx43 [34]. Therefore, the cytoskeletal connections between Cxs and integrins are thought to be important for the maintenance of normal protein distribution, expression, and/or functions of both groups of membrane molecules [8,34].

### 2.3. Functional Coupling Via Signaling Cascades

In contrast to the direct and indirect “physical” connections between Cxs and integrins, integrin-mediated signaling cascades are “chemically” coupled to the Cx opening via signaling cascades such as protein phosphorylation (Figure 1C). A good example of such chemical coupling has been observed in macrophages infected by the parasite *Entamoeba histolytica*, which activates integrin α5β1 [9]. Integrin activation in macrophages was associated with NLRP3 inflammasome activation and subsequent opening of the hemichannels comprised of pannexin-1, another channel-forming protein that shows a similar membrane topology to Cxs despite the lack of any sequence homologies between the two channel proteins [2]. The opening of the pannexin-1-containing hemichannel, which leads to ATP release into the extracellular space, was induced by the chemical modification (i.e., phosphorylation) of the pannexin-1 C-terminus via the integrin-mediated activation of the Src-family kinase. An alternative signaling cascade that links integrin activation to the opening of the hemichannel has been identified and involves integrin αVβ3. Ligand-induced activation of integrin αVβ3 induces the opening of Cx43 and pannexin-1 hemichannels, thereby giving rise to ATP release [35]. The integrin αVβ3-mediated PI3K-PLCγ-IP3R pathway eventually gives rise to Ca^2+^ release from the ER to the cytoplasm, triggering the opening of the hemichannel that, in turn, induces ATP release. Integrin-mediated ATP release via the hemichannel transactivates the P2X7 adenosine receptor, by which it is implicated in directed cell migration.

Several signaling cascades downstream of integrins have been shown to regulate Cx localization, membrane trafficking [27], and mRNA expression [32,36]. Integrin α3β1-mediated adhesion of keratinocytes to laminin, a major component of the basement membrane, not only strongly induced the formation of Cx43-containing gap junctions, but also promoted GJIC [27]. This integrin-mediated promotion of GJIC was shown to be mediated by RhoA signaling, which mediates intracellular Cx trafficking to support assembly of the gap junctions [27]. Integrin-linked kinase is one of the important adaptor proteins that mediates the downstream signaling following integrin-dependent cell adhesion [36]. Integrin-linked kinase activation in hepatocytes has been shown to induce the nuclear translocation of AKT, thereby leading to the suppression of Cx32 mRNA expression [36].

## 3. Connexins and Integrins in Exosomes

Exosomes are taken up by target cells, by which they are internalized in order to deliver any exosomal contents, including small molecules and small RNAs, to the cytoplasm [37,38]. Two major mechasnisms of exosomal uptake have been proposed: fusion to the cellular plasma membrane and endocytosis via several different modes (e.g., macropinocytosis, phagocytosis, clathrin-mediated endocytosis, caveolin-mediated endocytosis, and lipid-raft-mediated endocytosis) [37,38]. As described in the sections that follow, exosomal Cxs and integrins have been shown to play important roles in exosomal binding to, uptake by, and/or delivery of contents to, target cells.

### 3.1. Connexins in Exosomes

#### 3.1.1. Exosomal Connexin-Mediated Molecular Transfer to Target Cells

Girao and colleagues have demonstrated the presence of the Cx43 protein in the membranes of exosomes secreted by several cultured cell lines that express Cx43 on the cell surface [11]. Exosomes isolated from biological fluids and human plasma also contain the Cx43 protein. Exosomal Cx43 has been shown to assemble and attach to intact oligomers, thereby surviving to form hexameric hemichannels. Of note, exosomal Cx43-based hemichannels have been shown to be functionally active in their ability to transfer small molecules to target cells [11]. Exosomes expressing Cx43 exhibited the capability to transfer, to target cells, a dye luciferin (molecular weight 280) known to penetrate gap junction channels. The transfer of luciferin happens instantaneously, requiring only a few seconds. As the instantaneous nature of the transfer phenomenon is probably dissimilar to the mechanism underlying exosomal internalization, the transfer most likely occurs through a channel pore. Exosomal transfer of luciferin was inhibited by a peptide gap 26 that blocked Cx43-based channel functions [11]. In addition, exosomes containing mutant Cx43 (S368A), which inhibited serine phosphorylation, and thereby facilitated the formation of a constitutively open hemichannel, exhibited higher levels of instantaneous exosomal luciferin transfer [11]. By contrast, exosomes containing another mutant Cx43 (S368D), one that mimics serine phosphorylation at residue 368 and thereby facilitates the formation of a constitutively closed hemichannel, exhibited reduced levels of exosomal luciferin transfer. These results confirmed the Cx channel-dependent exosomal transfer of luciferin [11]. This transfer appears to take place through the docking of the exosomal hemichannel and via targeting of the cellular hemichannel, thus constituting a gap junction-like functional channel between exosomes and target cells [11]. The blocking peptide gap 26, which would disrupt the proper formation of Cx43-based hemichannels, inhibited the channel docking between exosomes and target cells [11].

In addition to the internalization of exosomes via fusion to the plasma membrane and endocytosis [37,38], this gap junction-like Cx channel-mediated mechanism might represent a novel alternative method for instantaneously delivering at least parts of the exosomal contents to target cells [11]. Cx channel-mediated transfer would precede, and/or simultaneously occur in tandem with, the uptake of exosomes via internalization. Thus, it is interesting to speculate that those small molecules instantaneously delivered via Cx channels might alter the metabolism of target cells, thereby preconditioning them to either promote or suppress subsequent exosomal uptake.

Another interesting point of speculation concerns the possibility that exosomes might form Cx43-based channels not only with target cells that endogenously express Cx43, but also with those cells that do not. As shown by the exosomal transfer of integrin proteins to target cells [22,39], the membrane fusion of Cx43-containig exosomes to target cells could lead to the incorporation of Cx43 into the plasma membranes of target cells. Therefore, the ability of exosomes to transfer the Cx43 protein could enable Cx43 channel-mediated exosomal delivery to virtually any target cell population, regardless of the endogenous expression of target cellular Cx43. This might entail a generalized model in which the Cx channel-mediated instantaneous delivery of exosomal contents occurs in tandem with exosomal uptake via internalization and membrane fusion during the course of ongoing intercellular exosomal communication.

#### 3.1.2. Cx-Mediated Transfer of Therapeutically and Biologically Active Exosomal Contents

Using a model involving molecular luciferin dye, Cx channel-mediated transfer of exosomal contents was shown to occur with proof-of-principal validity [11]. However, it remains to be elucidated exactly what type of therapeutically and/or biologically active exosomal contents would be delivered via the Cx channels to target cells.

A sequel study conducted by Girao and colleagues aimed to investigate the ability of exosomal Cx43 to enhance the delivery of the chemotherapeutic drug doxorubicin to tumors [40]. Although the presence of Cx43 on doxorubicin-loaded exosomes did not enhance the anti-tumor effects in vivo, exosomal Cx43 ameliorated the cardiotoxicity of doxorubicin [40]. Whereas the heart expresses Cx43, cardiac Cx43 is thought to locate mainly at the intercalated discs, forming gap junctions therein. Thus, cardiac Cx43 is not readily available for exosomal Cx43 to dock, thereby partly illustrating the molecular mechanism underlying exosomal Cx43-mediated amelioration of doxorubicin-induced cardiotoxicity, which remains unclear [40]. A possible additional explanation could be that Cx43-positive, doxorubicin-loaded exosomes are taken up by non-cardiac organs that express high levels of Cx43, such as the bone marrow, endocrine tissues, and skin [41].

MicroRNAs (miRNAs) are among the most biologically active components of exosomes, and have been extensively studied in terms of the exosome-induced functional alteration of target cells [42]. Notably, two RNA-binding motifs were predicted in the sequence of Cx43; specifically, at the cytoplasmic loop and C-terminal cytoplasmic regions [43]. Although the functions of RNA-binding motifs still await experimental testing, they are believed to facilitate the packing of small RNAs, including miRNAs, into Cx43-containing exosomes. Currently, the “gap junction-like” Cx channel-mediated exosomal miRNA transfer to target cells remains an attractive idea lacking experimental verification. By contrast, miRNA transfer through an authentic gap junction between cells has been demonstrated [44,45]. Mature miRNAs were transferred and exchanged via gap junctions between the cytoplasmic spaces of adjacent cells [45]. Gap junction-mediated exchange of mature miRNAs has been observed both between homotypic cells and between heterotypic cells [44]. For example, the miRNA exchange that occurs between endothelial cells and cancer cells has been demonstrated in vitro [44]. The physiological significance of gap junction-mediated miRNA exchanges between cells has yet to be elucidated; however, it may trigger bystander effects in gene therapy settings. In such cases, synthetic miRNA mimetics would be delivered not only onto the target cells, but also onto adjacent non-target cells [44]. The miRNA mimetics initially delivered to adjacent non-target cells could subsequently spread, reaching target cells through the gap junctions between adjacent and target cells [44]. In this way, gap-function-mediated bystander effects would enhance the therapeutic effects of miRNA mimetic treatments.

Kalluri and colleagues have demonstrated that cancer exosomes contain all of the necessary components for gene silencing to include miRNAs along with the RNA-induced silencing complexes (RISCs), which contain Dicer, Ago2, and trans-activating response RNA-binding proteins [46]. Thus, miRNA biogenesis (i.e., pri-miRNA processing by Dicer to transform into mature miRNA) [42] could occur within exosomes, which suggests they function somewhat like miRNA factories [47]. The simultaneous transfer of mature miRNAs into RISCs would enable gene silencing as soon as the exosomes were delivered to the target cells, independently of target cellular RISC components. These results need to be reproduced independently and the detailed nature of miRNA processing during exosomal biogenesis requires further and extensive investigation.

#### 3.1.3. Regulation of Exosome Release by Cellular Connexin

Several types of cellular stress (e.g., cellular senescence by irradiation [48]; endoplasmic reticulum stress by cisplatin [49]; and tunicamycin [50]) have been shown to increase the release of exosomes from cells. The regulatory role of Cx43 in exosome release has been shown in traumatic brain injury [51]. Cx43 in astroglias in the hippocampus has been implicated in propagating damage into surrounding brain tissues [51]. Using a rat model, Tong and colleagues have demonstrated that traumatic brain injury induced at the hippocampus results in the release of exosomes, which attempted to restore the injury-induced functional memory defects then present in the hippocampus neurons (i.e., long-term potentiation) [51]. Of note, Cx43 is responsible for promoting injury-induced exosomal release from the hippocampus [51], indicating its important role in both propagating and dampening neuronal damage in traumatic brain injury.

### 3.2. Integrins in Exosomes

#### 3.2.1. Integrin-Directed Exosomal Homing to Tissues and Remodeling of the Homing Niche

Integrins on leukocytes, such as α4β7 and αLβ2, support the critical adhesive interactions with their ligands MAdCAM-1 (Mucosal Addressin Cell Adhesion Molecule-1) and ICAM-1 (Intercellular Adhesion Molecule-1), respectively, on endothelial cells, thereby mediating migration to specific tissues [52]. This trafficking process is known as tissue-specific leukocyte migration (or homing). It constitutes an integral part of an effective adoptive immune response and of eliciting immune cell accumulation at the site of inflammation [52]. The concept of integrin-mediated cell migration to specific tissues, wherein endothelial cells express corresponding integrin ligands, can be generalized to the metastatic spread of cancer [53,54]. Some cancer cells are known to upregulate the expression of αVβ3, αVβ6, α5β1, and/or α6β4 [54]. These cancer-upregulated integrins bind to specific extracellular matrix protein ligands, such as vitronectin, fibronectin, laminin, and collagens, that are deposited in the cancer microenvironment, thereby promoting the metastatic dissemination of primary cancer cells to distant organs [54].

**Figure 2:**
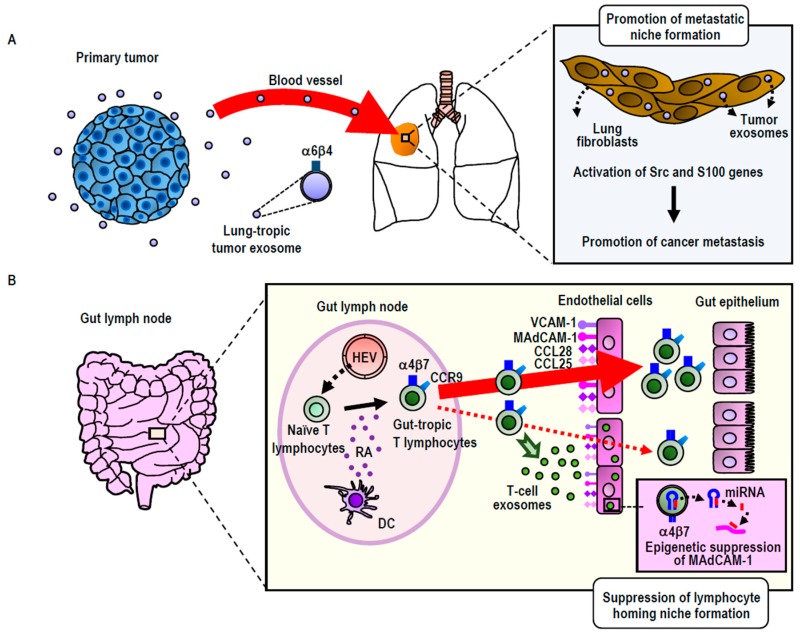
Exosomal remodeling of cancer metastatic (**A**) and lymphocyte homing (**B**) niches. (**A**) Integrin α6β4^high^ exosomes secreted by breast or pancreatic cancers preferentially distribute to the lung, wherein they remodel to promote metastatic niche formation via the activation of Src family kinases and S100 proinflammatory genes. (**B**) Integrin α4β7^high^ exosomes secreted by gut-tropic T-lymphocytes preferentially distribute to the gut, wherein they remodel to suppress homing niche formation by the down-regulation of the MAdCAM-1 gene via an miRNA-mediated epigenetic mechanism. HEV: High endothelial venules; RA: Retinoic acid; DC: Dendritic cells.

Exosomes express integrin on the surface. Consistent with the function of cellular integrins to guide cells to specific organs, exosomal integrins have been shown to be capable of guiding exosomes to specific tissues, as was originally shown in cancer exosomes. Using breast cancer and pancreatic cancer exosomes, Lyden and colleagues have shown that those exosomes expressing integrin α6β4 were preferentially distributed to the lung, whereas those expressing integrin αVβ5 were preferentially distributed to the liver [12]. It has been suggested that preferential exosomal distribution to the lung was mediated by the interaction of integrin α6β4 with laminin in the lung, whereas that to the liver was mediated by the interaction of integrin αVβ5 with fibronectin [12]. Interestingly, cancer exosomal homing to the lung and the liver does not simply precede the metastatic dissemination of primary breast and pancreatic tumors to these distant organs, but also preconditions the lung and liver to form pre-metastatic niches, a specialized microenvironment that promotes the homing, retention, and subsequent proliferation of disseminated cancer cells (Figure 2A). The proposed mechanism underlying pre-metastatic niche formation involves the activation of proinflammatory *S100* genes in lung and liver tissue cells [12,55]. This process is elicited by the Src activation activity carried out by exosomal integrin proteins transferred to target cells. Integrins themselves do not conduct kinase activities; instead, they associate with Src-family kinases, thereby acting as a hub for establishing signaling complexes [12]. While exosomal transfer of the intact integrin-signaling complex remains to be demonstrated, exosomally transferred integrin proteins could trigger the signals that lead to S100 activation by using Src kinases derived from either exosomes or target cells [12]. In this way, cancer exosomes establish the organotropism driving metastasis.

Building upon and extending the study of cancer exosomes to non-cancerous cells (i.e., T-lymphocytes), our group has utilized the concept of integrin-mediated exosomal homing to remodel the microenvironment of target tissues [22] (Figure 2B). Upon activation via contact with antigen-presenting dendritic cells residing in the gut, T-lymphocytes acquire gut-tropism—i.e., the ability to preferentially home to the gut by upregulating integrin α4β7 [56]. Integrin α4β7 binds to its endothelial ligand MAdCAM-1, which is exclusively and constitutively expressed in the gut, thereby playing a central role in gut-specific lymphocyte homing [56]. We have demonstrated that gut-trophic lymphocytes secrete exosomes expressing high levels of integrin α4β7, which guide the exosomes to the gut via binding to MAdCAM-1 [22]. In contrast to cancer exosomes, which promote the formation of pre-homing (or pre-metastatic) niches, gut-tropic T-lymphocytic exosomes diminish gut-homing niches by suppressing the expression of MAdCAM-1 and other homing-supporting molecules [22]. The ability of T-lymphocytic exosomes to diminish gut-homing niches has been attributed to several specific exosomal miRNAs that target those molecules, as well as the transcription factor that controls MAdCAM-1 expression [22]. These results underscore the significance of exosomal regulation to cell homing by modifying the microenvironments of destination tissues [22]. We propose that in certain physiological settings (i.e., non-cancerous cells), this exosomal regulation acts suppressively to balance the excessive accumulation of homed cells. However, in malignantly transformed cells, aberrant exosomal regulation could act in a promotive-like manner, thereby contributing to the pathogenesis of cancer metastasis.

#### 3.2.2. Integrin Protein Exosomal Transfer

Integrin-expressing exosomes have been shown to transfer integrin proteins to target cells [22,39]. Integrins exosomally transferred to target cells are found on the cell surface, possibly either via the direct membrane fusion of exosomes or via the internalization of exosomes that recycle integrins back to the membrane through early endosomes [22,39]. Prostate cancer cells express high levels of integrin αVβ3 and αVβ6, whereas normal prostate cells do not. In addition, the former secrete exosomes that express high levels of integrin αVβ3 and αVβ6, which facilitate the delivery of those integrins to other prostate cancer cells [22,39]. Target cells that take up the cancer exosomes may upregulate the cell-surface expression of integrin αVβ3 and αVβ6 [22,39]. Integrin upregulation in target cells was induced without any increase in mRNA levels in the case of αV integrins, evidence that supports the direct transfer of exosomal integrin proteins to target cells [22,39]. The resultant integrin upregulation leads to the enhancement of cell adhesion and migration on those substrates containing αV integrin ligands [22,39]. When the exosomal αV integrin transfer occurs from αV integrin^high^ aggressive cancer cells to αV integrins^null^ benign hyperplasic cells, the latter acquire the capability to exhibit αV integrin-mediated aggressive migratory behavior. Thus, an important functional consequence of the exosomal transfer of αV integrins is the horizontal transmission of the ability to migrate aggressively [22,39].

Another important functional consequence of the exosomal transfer of αV integrins is the horizontal transmission of the ability to activate TGF-β signaling. TGF-β is a cytokine that demonstrates an array of immunosuppressive, anti-inflammatory, and pro-fibrotic activities. The biogenesis of active TGF-β is unique in its requirement for integrin-mediated force [57]. TGF-β is produced and secreted to the extracellular space as an inactive precursor in which mature TGF- β is caged in LAP (latency-associated protein), thereby confining its bioactivities [57]. LAP contains an integrin-binding RGD motif, which allows αV integrins (e.g., αVβ3, αVβ5, αVβ6, and αVβ8) to bind and impose a mechanical force to open the LAP cage. This leads to the freeing of the mature active TGF-β into the microenvironment, which increases the local concentration of TGF-β and enables its binding to TGF-β receptors expressed on neighboring cells [57]. The exosomally transferred αVβ6 protein has been shown to activate TGF-β in target cells [58]. In this study, the exosomal transfer of integrin αVβ6 and the resulting horizontal transmission of TGF-β-activating ability was investigated in the context of immune response regulation, specifically immune tolerance to food antigens in the gut [58]. Gut epithelial cells secrete exosomes containing αVβ6 and cognate intestinal food antigens, by which the αVβ6 integrin and antigen are transferred to mucosal dendritic cells. This enables dendritic cells to express αVβ6, and thereby activate TGF-β in the surrounding microenvironment. These actions allow the T cell-dendritic cell interactions to occur while being exposed to TGF-β [58]. T-cell activation in the presence of TGF-β induces the differentiation to regulatory T cells, which induces tolerogenic responses to food antigens.

In addition, intracellular signaling proteins associated with integrin cytoplasmic domains in parent source cells might be contained in exosomes, and could thereby be transferred to target cells. As mentioned earlier in this section [12], certain Src-family kinases associated with cytoplasmic integrin domains are believed to undergo co-transference with αV-integrins by exosomes to target tissues, thus giving rise to the Src-dependent induction of proinflammatory *S100* genes [12,55]. However, it remains to be elucidated whether such Src-family kinases are physically complexed with integrins in exosomes, or if kinases and integrins merely co-exist therein. The co-existence of integrins and important integrin adopter proteins, such as talin and vinculin, has already been demonstrated [59], whereas the physical association with integrins in exosomes has yet to be reported. We have recently shown functional evidence that talin physically associates with integrins in exosomes, in this way regulating the activity of exosomal integrins to bind ligands. How well the integrin signaling adaptor protein complexes formed in the cell are maintained in exosomes; whether the complexes are successfully transferred to target cells; and, if transferred, what functional roles they might play in target cells, remain significant questions to be addressed in the future.

#### 3.2.3. Autocrine Roles Played by Exosomes in the Regulation of Cell Migration

In addition to being involved in intercellular communication with neighboring and distant cells, exosomes also act in an autocrine signaling-like manner, in which cellular and exosomal integrins cooperate in a very precise regulation of directional cell movement within the interstitial space [60]. Integrin α5β1 expressed on fibrosarcoma cells takes up and internalizes soluble fibronectin from the extracellular space [60,61]. Internalized fibronectin is processed in the endosomal pathway and then transferred to the exosome biogenesis pathway, in which fibronectin is captured by the exosomal integrin α5β1 [60]. In this way, fibronectin-bound integrin α5β1-expressing exosomes are produced, before being secreted to the extracellular space surrounding the cell. These fibronectin-bound integrin α5β1-expressing exosomes are then locally deposited on collagen fibers in the interstitial space, thereby paving the way, with fibronectin, for cellular integrin α5β1-mediated adhesion and the migration of fibrosarcoma cells [60]. The plasma-membrane microregion, where exosomes are secreted, establishes a point of stable integrin-mediated adhesion to the fibronectin deposited in the interstitial space, which determines the direction of cell migration [60]. The region that is home to this exosome secretion subsequently becomes a part of the protruding leading edge (i.e., front) of migrating cells, while enhancing the secretion of exosomes from nearby regions. This positive feedback loop, which involves the autocrine effects of integrin-expressing exosomes, supports persistent directional cell migration [60].

#### 3.2.4. Exosomal Integrin as a Biomarker

Altered expression of integrin subsets on exosomes circulating in the blood has been reported in cancer and non-cancerous inflammatory disorders. In patients suffering from metastatic lung cancers, the levels of β4 integrin in exosomes in the plasma were significantly elevated, regardless of the origin of their cancers [12]. In contrast, in patients who have liver metastasis, the levels of αV integrin in exosomes in the plasma were significantly elevated. These results were in good agreement with the in vivo results showing that α6β4- and αVβ5-expressing cancer exosomes were preferentially distributed to the lung and the liver, respectively, wherein the remodeling of tissues to form premetastatic niches occurs.

We have studied the levels of integrin expression on exosomes in the plasma of sepsis patients admitted to an ICU (Intensive Care Unit) [62]. Sepsis is a type of life-threatening generalized inflammation induced by aberrantly activated immune responses to infection [63]. Whereas the exosomal expression levels of β1 and β3 integrins were comparable between sepsis patients and healthy subjects, that of β2 integrin was significantly increased in sepsis patients [62]. In addition, levels of exosomal β2 integrin expression in sepsis patients correlated well with the severity of circulatory collapse and kidney failure. As β2 integrins are only expressed in leukocytes, increased exosomal β2 integrin expression is thought to reflect the enhanced systemic activation of leukocytes, which could lead to inflammatory tissue injury [62].

## 4. Potential Cross-Talk of Integrins with Connexins in Exosomes

As it is assumed that integrins and Cxs are simultaneously present in at least some exosomes, we decided to discuss the potential cross-talk between integrins and Cxs in exosomes, as has been described in cells [7,8,9]. First, a direct physical association of integrins with Cxs in the membrane might be maintained in some exosomes (Figure 1A). As is shown in cells [7], this direct coupling of integrin conformational changes to the Cx opening might enable Cx channel openings upon ligand binding by integrin. Such integrin-Cx couplings would allow for the rapid transfer of exosomal contents through Cx channels to target cells upon the integrin-mediated binding of exosomes to the surface of target cells. Secondly, integrins and Cxs might be physically inter-connected via actin in exosomes (Figure 1B). In cells, integrins are connected to actin via talin or vinculin, while Cxs are connected to actin via the ZO-1 and vinculin complex, thereby supporting the inter-connection of integrins and Cxs by actin [8]. These proteins, which are essential to inter-connect integrins and Cxs, are assumed to be incorporated through the endosomal pathway to exosomes. The association of talin with integrins in exosomes is thought to up-regulate both ligand binding and subsequent integrin-mediated internalization to target cells. Therefore, actin-mediated coupling of Cxs with integrins might be involved in the regulation of exosomal functionalities related to adhesion and internalization by target cells. Third, integrin-mediated signaling might modify the opening of Cx channels in exosomes (Figure 1C). Exosomal integrins have been shown to carry Src-family kinases, which might induce the phosphorylation of a key tyrosine residue of Cxs, thereby modifying the opening/closing mechanism of Cx channels in exosomes. Alternatively, Src might be associated with exosomal Cxs, as Src has been shown to bind to, and induce the tyrosine phosphorylation of, Cx43, thereby disrupting the Cx43-mediated GJIC in transformed cells [64]. This process might alter the efficacy of the instantaneous exosomal transfer of bioactive molecules through Cx channels to target cells. These intriguing possibilities surrounding the cross-talk between integrins and Cxs in exosomes warrant future experimental study.

## 5. Conclusions

Recent investigations have begun to reveal the important roles of Cxs and integrins in exosomes. Exosomal Cxs support docking to target cells, thus aiding a novel mechanism to transfer exosomal contents to the cells. Exosomal integrins support targeted tissue distributions, and in this manner, remodel the microenvironment following the migration of cancer and immune cells. In addition, the exosomal transfer of Cxs, integrins, and their associated proteins such as Src, could alter the functions and metabolism of target cells. Furthermore, Cx-integrin crosstalk, which has been shown to occur in at least three different mechanisms in cells, might operate in exosomes, potentially orchestrating the pathogenesis of cancer and inflammation.
